# Fewer Pills, Lower Bills: Antihypertensive and Cost Outcomes of Adrenalectomy in Primary Aldosteronism

**DOI:** 10.1245/s10434-026-19347-0

**Published:** 2026-03-04

**Authors:** Jesse E. Passman, Lily Owei, Colleen Brensinger, Taryn Barrett, Lauren R. Kelz, Jasmine Hwang, Jordana B. Cohen, Heather Wachtel

**Affiliations:** 1 Department of Surgery, Hospital of the University of Pennsylvania, Philadelphia, PA USA; 2 Leonard Davis Institute, University of Pennsylvania, Philadelphia, PA USA; 3 The Center for Clinical Epidemiology and Biostatistics, Perelman School of Medicine of the University of Pennsylvania, Philadelphia, PA USA; 4 Renal-Electrolyte and Hypertension Division, Department of Medicine, Perelman School of Medicine, University of Pennsylvania, Philadelphia, PA USA; 5 Perelman School of Medicine, University of Pennsylvania, Philadelphia, PA USA

**Keywords:** Primary aldosteronism, Adrenalectomy, Hypertension, Aldosterone, Endocrine, Resistant hypertension, Hypokalemia

## Abstract

**Background:**

Primary aldosteronism (PA) can be treated surgically or medically depending on disease lateralization and surgical candidacy. There is a dearth of data directly comparing antihypertensive medication trajectories and costs between these strategies.

**Patients and Methods:**

We performed a retrospective cohort study of patients with new PA diagnoses and adrenal vein sampling to assess antihypertensive medication outcomes and treatment costs using Optum’s de-identified Clinformatics^®^ Data Mart Database (2004–2022). Patients were stratified by receipt of adrenalectomy versus medical management alone. The index time point was defined as adrenal vein sampling (AVS) for medically managed and adrenalectomy for surgically managed patients. Outcomes were assessed using regression models.

**Results:**

Of 911 patients, 52% underwent adrenalectomy and 48% medical therapy. Adrenalectomy patients were younger, with higher Elixhauser scores. Antihypertensive medication use (2.9 versus 2.8, *p* = 0.636) and costs did not differ at index. After 1 year, adrenalectomy patients used fewer antihypertensive medications (1.5 ± 1.4) than medically managed patients (2.5±1.5, *p* < 0.001). On regression, age (*β* = 0.02, *p* = 0.002), male sex (*β* = 0.40, *p* < 0.001), and baseline antihypertensive medications (*β* = 0.43, *p* < 0.001) were associated with higher antihypertensive medication requirement. Adrenalectomy patients were prescribed 1.11 fewer antihypertensive medications at one year (*p* < 0.001). In the resistant hypertension subcohort, adrenalectomy reduced antihypertensive medications by 1.35 (*p* < 0.001). Adrenalectomy was associated with US $908 lower antihypertensive medication prescription costs (*p* < 0.001) and 87% lower odds of potassium supplementation (*p* < 0.001).

**Conclusions:**

Patients with PA who undergo adrenalectomy demonstrate a significant reduction in antihypertensive medications compared with medically managed patients. While there is significant upfront cost to surgical intervention, reduced long-term prescription costs are realized.

**Supplementary Information:**

The online version contains supplementary material available at 10.1245/s10434-026-19347-0.

Primary aldosteronism (PA) is the leading cause of secondary hypertension, accounting for an estimated 5–18% of all cases of hypertension.^1^ However, it remains grossly underdiagnosed, leading to untoward morbidity in a significant population.^2^ Compared with essential hypertension, PA is associated with significantly higher rates of adverse cardiovascular, renal, and metabolic outcomes.^3^ PA results from autonomous aldosterone production, most commonly due to a benign aldosterone-secreting adrenal adenoma or adrenal hyperplasia.^4^ Approximately one third of cases are attributable to a unilateral source, for which adrenalectomy is the treatment of choice and can be curative.^5^ The remaining two thirds of patients with PA typically have PA due to bilateral adrenal hyperplasia.^6^ These patients, as well as those medically unfit for surgery, are typically treated with mineralocorticoid receptor antagonists (MRAs), such as spironolactone and eplerenone.^7,8^ Adrenal vein sampling (AVS) is the gold standard test for distinguishing unilateral from bilateral disease when surgical management is being considered.^6,9^

In patients with unilateral causes of PA, adrenalectomy is the preferred approach given its excellent clinical outcomes and low morbidity.^10^ Adrenalectomy results in biochemical cure in 96–100% of patients.^11^ Clinical cure—defined as normotension without the need for antihypertensive medications—occurs in approximately 50% of patients, with a significant number having an ongoing, although decreased, requirement for antihypertensive medications after adrenalectomy.^12^ Adrenalectomy also reduces the risk of cardiovascular morbidity independent of blood pressure control.^6^ In contrast, patients managed medically typically need lifelong therapy, with many patients requiring additional antihypertensive medications to achieve adequate blood pressure control.^11,13^

Despite robust evidence supporting surgical management in appropriately selected patients, few studies have directly quantified and compared the relative antihypertensive medication burden, nor the difference in healthcare costs between surgical and medical management strategies.^14^ This study aims to: (1) quantify trends in antihypertensive medication use among patients with PA treated with adrenalectomy versus medical therapy and (2) compare the direct healthcare costs associated with surgical and medical management over time.

## Patients and Methods

### Data Source—Optum Clinformatics^®^

We performed a retrospective cohort study utilizing Optum’s de-identified Clinformatics^®^ Data Mart Database (Optum^®^ CDM or Optum Clinformatics^®^) to evaluate outcomes for patients diagnosed with PA from 2004 to 2022.

Optum Clinformatics^®^ is derived from a database of administrative health claims for members of large commercial and Medicare Advantage health plans, capturing claims data from more than 77 million patients. Optum Clinformatics^®^ uses medical and pharmacy claims to derive patient level enrollment information, healthcare costs, and resource utilization information. The population is geographically diverse, spanning all 50 states, and is statistically de-identified under the HIPAA Privacy Rule’s Expert Determination method and managed according to Optum^®^ customer data use agreements. Optum^®^ CDM administrative claims are submitted for payment by providers and pharmacies are verified, adjudicated, and de-identified prior to inclusion.

This study was deemed exempt from review by the Institutional Review Board of the University of Pennsylvania. Strengthening the Reporting of Observational Studies in Epidemiology (STROBE) guidelines were followed throughout Supplementary Information 1.^15^

### Study Cohort

**Fig. 1:**
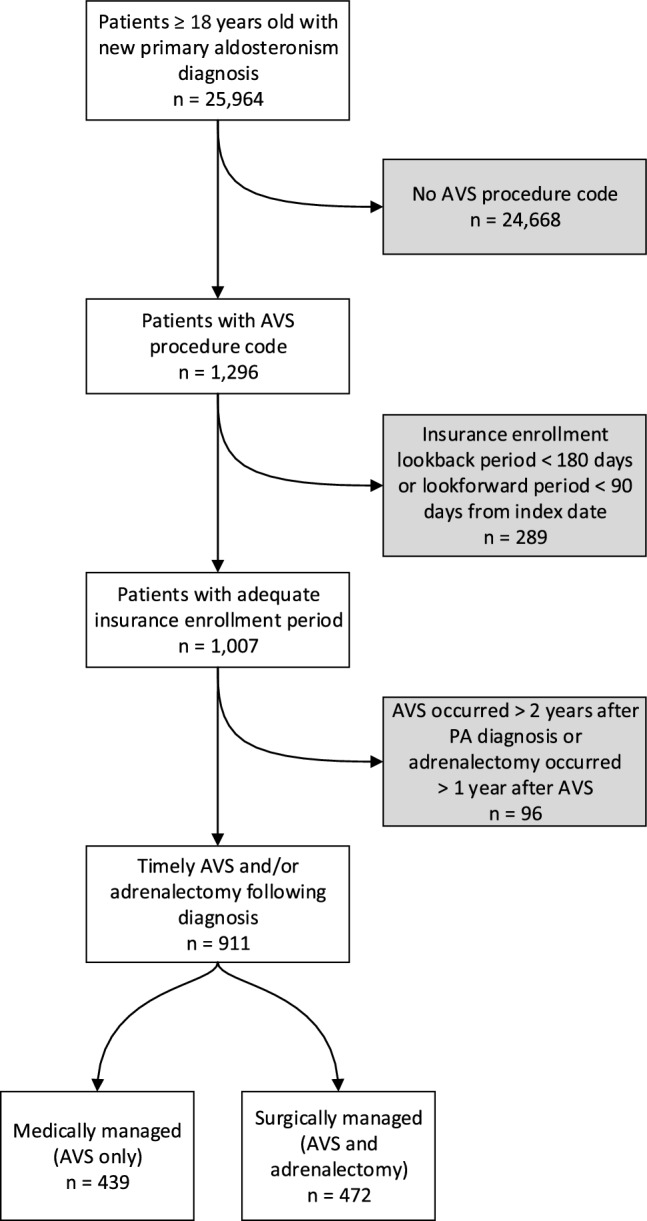
CONSORT diagram

The initial cohort that was screened for inclusion included patients ≥ 18 years of age who had an incident PA diagnosis as identified by ICD code (Supplementary Information 2) between 2004 and 2022. To ensure appropriate diagnostic approach, patients who did not receive adrenal vein sampling (AVS) within 2 years were then excluded. Patients were then stratified by receipt of adrenalectomy within 1 year of AVS, creating two groups: (1) patients who underwent adrenalectomy or (2) patients who received medical management only. Patients were excluded if they did not have at least 6 months of data prior to diagnosis and 3 months of data following intervention, representing lapses in insurance coverage not captured by the database. The final cohort included 911 patients (Fig. 1). Subgroup analyses were performed on a cohort of patients with apparent treatment resistant hypertension, defined by utilization of ≥ 3 antihypertensive medications at index.

### Outcomes of Interest

The primary outcomes were: (1) antihypertensive medication requirements and (2) adjusted costs of treatment. Antihypertensive medications were grouped by class (Supplementary Information 3), with the number of antihypertensive medications received by the patient defined as the number of different antihypertensive classes prescribed within each allotted time period. Combination medications were identified manually within the dataset and assigned to multiple classes as appropriate. Index date was defined as “date of adrenalectomy” in surgically managed patients versus “date of AVS” within nonoperatively managed patients.

Cost analysis examined adjusted standard costs (all medical claims from date of admission to date of discharge) for operatively managed patients, adjusted medical costs (costs for 30 days after discharge including medical and prescription claims), and cumulative antihypertensive medication prescription costs. Costs of antihypertensive medications were directly compared between surgically and medically managed cohorts at set periods preceding and following index date. Costs were adjusted by year and type of service.

An additional subanalysis was performed evaluating discontinuation of potassium supplementation. Potassium supplementation was considered a binary variable with supplements defined by their generic names (Supplementary Information 4).

### Variables

Other variables included age, sex, race, education level, insurance status, household income, and Elixhauser score. Race, education level, and household income were stratified in accordance with the Optum Clinformatics^®^ categories. Insurance status was either commercial/private or Medicare.

### Statistical Analysis

Descriptive statistics were tabulated. Categorical variables were expressed using frequencies and percentiles and continuous variables were expressed as mean and standard deviation (SD) for normally distributed variables or as median and interquartile range (IQR) for non-normally distributed variables. Group comparisons were performed for the primary outcomes, stratified by surgical versus medical management using, Student’s *t*-tests, Wilcoxon rank-sum tests, and *χ*^2^ tests, as appropriate. Multivariable linear regression models assessing number of antihypertensive medications and prescription costs at 1-year post index were performed with covariates including receipt of adrenalectomy, age, sex, race, ethnicity, household income, insurance status, and Elixhauser score. The baseline number of antihypertensive medications or cost of antihypertensive medications at index time point were included in the respective models. Analysis was conducted with SAS^®^, version 9.4.^16^ A *p*-value of < 0.05 was regarded as statistically significant.

Subgroup analysis was performed analyzing antihypertensive medication outcomes and costs in patients with resistant hypertension, defined by utilization of ≥ 3 antihypertensive medications at index, in the same fashion as the primary analysis. Additional subgroup analysis evaluated rates of potassium supplementation at index versus at 6 months post index.

## Results

### Cohort Characteristics

In total, 911 patients met inclusion criteria; 472 were managed surgically, and 439 were managed medically. Key cohort demographics and characteristics can be found in Table 1. Patients who underwent surgical management tended to be younger (median age 55.0 versus 57.0 years, *p* = 0.003) with significantly more Elixhauser comorbidities (median 1.0 versus 0.0, *p* < 0.001). A greater proportion of surgically managed patients lived in higher income households with 34.5% having a household income ≥ US $100,000 versus 29.4% of medically managed patients.

**Table 1:** Baseline demographic characteristics comparing patients medically versus surgically managed

Variable	Overall cohort (*n* = 911)	Medically managed (*n* = 439)	Adrenalectomy (*n* = 472)	*p*-Value^b^
Median age, years (IQR)^a^	55.0 (47.0–64.0)	57.0 (48.0–65.0)	55.0 (47.0–64.0)	**0.003**
Sex				
Female	359 (39.4%)	187 (42.6%)	172 (36.4%)	0.057
Male	552 (60.6%)	252 (57.4%)	300 (63.6%)	
Race				
Asian	36 (4.0%)	15 (3.4%)	21 (4.4%)	0.370
Black	160 (17.6%)	85 (19.4%)	75 (15.9%)	
Hispanic	89 (9.8%)	48 (10.9%)	41 (8.7%)	
White	571 (62.7%)	267 (60.8%)	304 (64.4%)	
Unknown	55 (6.0%)	24 (5.5%)	31 (6.6%)	
Education level				
Less than 12th grade	*N* < 5	*N* < 5	*N* < 5	0.492
High school diploma	189 (20.7%)	93 (21.2%)	96 (20.3%)	
Less than bachelor degree	487 (53.5%)	238 (54.2%)	249 (52.8%)	
Bachelor degree plus	196 (21.5%)	90 (20.5%)	106 (22.5%)	
Unknown	36 (4.0%)	18 (4.1%)	18 (3.8%)	
Household income				
< US $40K	165 (18.1%)	72 (16.4%)	93 (19.7%)	**0.039**
US $40,000–49,000	48 (5.3%)	29 (6.6%)	19 (4.0%)	
US $50,000–59,000	52 (5.7%)	33 (7.5%)	19 (4.0%)	
US $60,000–$74,000	90 (9.9%)	39 (8.9%)	51 (10.8%)	
US $75,000–$99,000	132 (14.5%)	69 (15.7%)	63 (13.3%)	
US $100,000+	292 (32.1%)	129 (29.4%)	163 (34.5%)	
Unknown	132 (14.5%)	68 (15.5%)	64 (13.6%)	
Insurance status				
Commercial	690 (75.7%)	321 (73.1%)	369 (78.2%)	0.075
Medicare	221 (24.3%)	118 (26.9%)	103 (21.8%)	
Elixhauser score				
Median (IQR)	1.0 (0.0-4.0)	0.0 (0.0-2.0)	1.0 (0.0-4.0)	**< 0.001**
Mean (SD)	2.3 (2.7)	1.5 (2.6)	2.9 (2.6)	**< 0.001**

Bold values indicates *p *< 0.05

^a^Age at index date

^b^*p*-Value compares medically managed versus adrenalectomy patients

### Antihypertensive Usage Trajectories

**Fig. 2:**
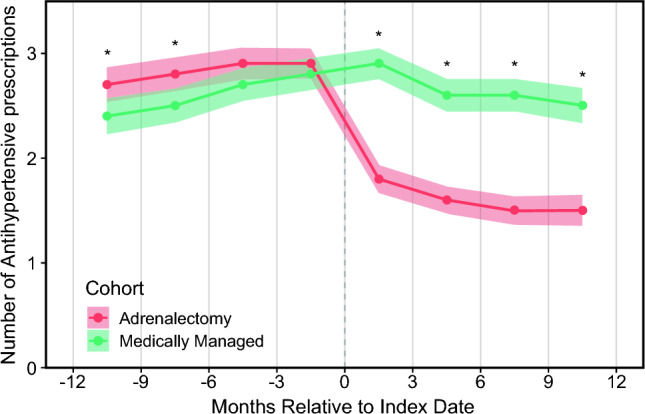
Trends in antihypertensive medications from pre-index to post index; ribbons represent 95% confidence intervals for each cohort; asterisks indicate *p* < 0.05 between cohorts at that time point

Quantities of prescribed antihypertensive medications were compared between the medically and surgically managed cohorts over 3-month time periods. There was a general upward trend in the number of antihypertensive medications in the year preceding the index time point for both cohorts (Fig. 2). While the surgically managed patients had a significantly higher number of prescribed antihypertensive medications between 9 and 12 months before index (2.7 versus 2.4, *p* = 0.003), and 6–9 months before index (2.8 versus 2.5, *p* = 0.024), there was no difference between number of antihypertensive medications in the 6 months preceding index (Table 2 and Fig. 2). After index, both cohorts saw a downward trend in the number of prescribed antihypertensive medications, with the largest drop occurring right after index time point. Patients undergoing adrenalectomy were prescribed a significantly lower number of antihypertensive medications at each time point; by 9–12 months post index, surgically managed patients were prescribed 1.0 fewer antihypertensive medications (1.5 versus 2.5, *p* < 0.001).

**Table 2:** Trends in mean number of prescribed antihypertensive medication classes from pre- to post-index date

Time period	Overall cohort		Medically managed		Adrenalectomy		*p*-Value^a^
	*n*	Mean (SD)	*n*	Mean (SD)	*n*	Mean (SD)	
Prior to index date							
−12 to −9 months	782	2.6 (1.7)	377	2.4 (1.7)	405	2.7 (1.7)	**0.003**
−9 to −6 months	844	2.7 (1.7)	413	2.5 (1.7)	431	2.8 (1.7)	**0.024**
−6 to −3 months	911	2.8 (1.7)	439	2.7 (1.7)	472	2.9 (1.7)	0.120
−3 to 0 months	911	2.8 (1.6)	439	2.8 (1.6)	472	2.9 (1.6)	0.636
After index date							
0 to +3 months	911	2.3 (1.6)	439	2.9 (1.6)	472	1.8 (1.5)	**< 0.0001**
+3 to +6 months	826	2.1 (1.6)	393	2.6 (1.6)	433	1.6 (1.4)	**< 0.0001**
+6 to +9 months	735	2.0 (1.6)	342	2.6 (1.5)	393	1.5 (1.4)	**< 0.0001**
+9 to +12 months	653	2.0 (1.5)	306	2.5 (1.5)	347	1.5 (1.4)	**< 0.0001**

Bold values indicates *p *< 0.05

^a^*p*-Value compares medically managed versus adrenalectomy patients

### Regression Modeling for Antihypertensive Burden 1 Year Post Index

Regression modeling was performed to control for confounders affecting antihypertensive medication burden at 9–12 months post index (Table 3). Increasing age (*β* = 0.02, 95% confidence intervals (CI) 0.01–0.03, *p* = 0.002), male sex (*β* = 0.40, 95% CI 0.20–0.60, *p* < 0.001), Black race (*β* = 0.27, 95% CI 0.00–0.54, *p* = 0.048), and higher baseline antihypertensive medication requirement (*β* = 0.43, 95% CI 0.37–0.49, *p* < 0.001) were associated with higher antihypertensive medication burden at 1 year. Adrenalectomy was significantly associated with lower number of prescribed antihypertensive medication classes at one year (*β* = −1.11, 95% CI −1.31 to −0.91, *p* < 0.001).

**Table 3:** Multivariable linear regression modeling analyzing predictors of increased antihypertensive burden after 12 months

Covariate	Coefficient	95% CI	*p*-Value
Age, per year	0.02	(0.01, 0.03)	**0.002**
Male sex	0.40	(0.20, 0.60)	**< 0.001**
Race/ethnicity (ref: white)			
Asian	−0.09	(−0.58, 0.39)	0.700
Black	0.27	(0.00, 0.54)	**0.048**
Hispanic	0.09	(−0.22, 0.41)	0.557
Unknown	0.13	(−0.31, 0.56)	0.559
Household income (ref: $100,000+)			
< US $40,000	0.00	(−0.29, 0.30)	0.983
US $40,000–$49,000	−0.31	(−0.75, 0.13)	0.165
US $50,000–$59,000	0.27	(−0.17, 0.70)	0.228
US $60,000–$74,000	−0.15	(−0.48, 0.18)	0.364
US $75,000–$99,000	−0.14	(−0.45, 0.17)	0.383
Unknown	0.05	(−0.28, 0.37)	0.770
Medicare insurance (ref: commercial)	−0.28	(−0.57, 0.01)	0.055
Elixhauser score, per point	0.01	(−0.03, 0.05)	0.642
Baseline number of antihypertensive medication, per antihypertensive medication	0.43	(0.37, 0.49)	**< 0.001**
Adrenalectomy	−1.11	(−1.31, −0.91)	**< 0.001**

Bold values indicates *p *< 0.05

### Perioperative and Antihypertensives Costs

Total adjusted standardized admission costs were tabulated for patients undergoing adrenalectomy. The median cost was US $26,805.50 (IQR US $12,802.37–31,816,81). The median cost of all claims within 30 days of discharge was $570.37 (IQR $260.09–1,786.12).

**Fig. 3:**
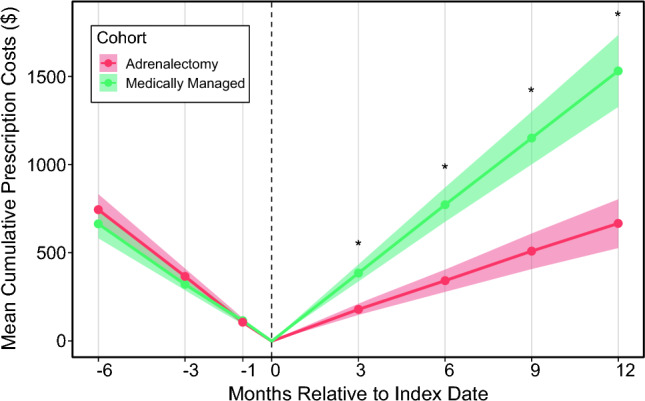
Trends in mean cumulative prescription costs over time in medically and surgically managed patients; ribbons represent 95% confidence intervals for each cohort; asterisks indicate *p* < 0.05 between cohorts at that time point

Antihypertensive medication prescription costs were tabulated in the time preceding and following the index date for each patient (Fig. 3 and Supplementary Information 5). The median prescription costs in the preceding 6 months across the cohort was US $313 (IQR US $89–1006). There was no significant difference in these costs between the surgically and medically managed cohorts. Post index, patients managed with adrenalectomy demonstrated significant decreases in prescription costs. By 1 year, these patients had incurred US $669 lower costs overall.

### Regression Modeling for Prescription Medication Costs 1 Year Post Index

Regression modeling was used to assess for variables associated with decreased prescription costs at 1 year (Table 4). Increasing age (*β* = 12.77 per year, 95% CI 1.43–24.12, *p* = 0.027), male sex (*β* = 590.79, 95% CI 361.10–820.48, *p* < 0.001), commercial insurance (*β* = 438.21, 95% CI 106.92–769.50, *p* = 0.010), and higher baseline antihypertensive medication prescription costs (*β* = 2.88 per dollar, 95% CI 2.37–3.40, *p* < 0.001) were associated with higher cumulative antihypertensive medication prescription costs at 1 year. Adrenalectomy was significantly associated with lower cumulative prescription costs at 1 year (*β* = −908.34, 95% CI −1134.86 to −681.81, *p* < 0.001).

**Table 4:** Multivariable linear regression modeling analyzing predictors of increased prescription costs over 12 months

Covariate	Coefficient	95% CI	*p*-Value
Age, per year	12.77	(1.43, 24.12)	**0.027**
Male sex	590.79	(361.10, 820.48)	**< 0.001**
Race/ethnicity (ref: white)			
Asian	−242.44	(−800.43, 315.55)	0.394
Black	181.85	(−126.80, 490.49)	0.248
Hispanic	19.77	(−338.53, 378.07)	0.914
Unknown	26.69	(−473.93, 527.30)	0.917
Household income (ref: US $100,000)			
< US $40,000	17.62	(−320.88, 356.13)	0.919
US $40,000–$49,000	−351.81	(−858.26, 154.64)	0.173
US $50,000–$59,000	124.14	(−376.24, 624.52)	0.626
US $60,000–$74,000	−131.78	(−509.79, 246.23)	0.494
US $75,000–$99,000	−11.45	(−366.78, 343.89)	0.950
Unknown	177.70	(−194.65, 550.05)	0.349
Medicare insurance (ref: commercial)	−438.21	(−769.50, −106.92)	**0.010**
Elixhauser score, per point	−1.13	(−43.90, 41.65)	0.959
Baseline monthly antihypertensive medication cost, per US$	2.88	(2.37, 3.40)	**< 0.001**
Adrenalectomy	−908.34	(−1134.86, −681.81)	**< 0.001**

Bold values indicates *p *< 0.05

### Rates of Potassium Supplementation

At the index time point, 52% (*n* = 476) of patients were prescribed potassium supplementation; patients who underwent adrenalectomy had a higher overall rate of supplementation (60% versus 44%, *p* < 0.001) (Supplementary Information 6). By 12 months post index, 13% (*n* = 82) of patients were taking potassium supplementation. Surgically managed patients had significantly lower rates of potassium supplementation at this time point (4.6% versus 22%, *p* < 0.001). On regression, patients prescribed potassium at baseline had over four times the odds of requiring potassium supplementation at 12 months (odds ratio, OR = 4.05, 95% CI 2.27–7.21, *p* < 0.001). Patients who underwent adrenalectomy demonstrated 87% lower odds of need for potassium supplementation compared to those who were medically managed (OR = 0.13, 95% CI: 0.07–0.24, *p* < 0.001) (Supplementary Information 7).

### Patients with Resistant Hypertension

A subcohort analysis was performed, assessing the rates of antihypertensive medication use and costs in a subcohort of patients with resistant hypertension. This subcohort consisted of 534 patients, of whom 251 (47%) were managed surgically and 283 (53%) medically (Supplementary Information 8). In this subcohort, surgically managed patients were slightly younger (median 57.0 versus 58.0 years, *p* = 0.009) with higher median Elixhauser scores (2.0 versus 0.0, *p* < 0.001). Similar to the overall cohort, patients with resistant hypertension who underwent adrenalectomy were prescribed a lower mean number of antihypertensive medications than medically managed patients at all time points post index (1 year: 1.9 (±1.5) versus 3.3 (±1.2), *p* < 0.001) despite similar baseline requirements (3.9 versus 4.0, *p* = 0.680) (Supplementary Information 9). This translated to significant cumulative antihypertensive medication cost savings at 1 year ($326.03 versus $1335.95, *p* < 0.001) (Supplementary Information 11).

Multivariable regression demonstrated increased age, male sex, Black race, and baseline number of antihypertensive medications were associated with higher antihypertensive medication use at 1 year. Adrenalectomy was associated with 1.35 lower antihypertensive medications at 1 year (*p* < 0.001) (Supplementary Information 10). Regarding cost, male sex, and baseline antihypertensive medication cost were associated with increased cumulative antihypertensive medication costs at 1 year; adrenalectomy was associated with US $1050.60 lower costs (95% CI 734.56–1366.63, *p* < 0.001) (Supplementary Information 12).

The median cost of adrenalectomy among patients with resistant hypertension was US $26,870.27 (IQR US $13,001.65–31,943.15), with a median cost of all claims within 30 days of discharge of US $616.76 (IQR US $307.02–2,019.34), similar to the overall cohort.

### Medically Managed Patients Receiving Directed Therapy

A subcohort analysis was performed comparing patients who underwent adrenalectomy versus those who underwent medical therapy which included an MRA. Prior to the index date, 303 (64%) surgically managed patients were prescribed an MRA compared with 225 (51%) medically managed patients. Post index, 77 (16%) surgically managed and 363 (83%) medically managed patients were prescribed an MRA. Adrenalectomy patients were prescribed fewer mean antihypertensive medications (1.5 versus 2.8, *p* < 0.001) and incurred fewer prescription costs at 1 year (US $665.76 versus US $1713.97, *p* < 0.001) than patients who were treated medically with an MRA, despite similar baseline values. On regression, adrenalectomy was associated with 1.25 fewer antihypertensive medications (95% CI 1.04–1.46, *p* < 0.001) and US $1045 in cost savings (95% CI US $803.52–1287.29, *p* < 0.001) than medical management including an MRA.

## Discussion

In this study, we quantified and compared surgical and management strategies for treatment of PA. We found that while medically and surgically managed patients do not differ in their baseline antihypertensive medication burden or prescription costs, patients managed surgically demonstrated significantly improved outcomes, with fewer prescribed antihypertensive medications at 1 year and prescription cost savings of about US $900 over that time period. Additionally, surgically managed patients demonstrated lower rates of potassium supplementation at 6 months.

Our findings align with prior studies evaluating the cost effectiveness of adrenalectomy compared to medical management of PA.^14,17^ Prior simulation studies have found that screening, diagnosis, and treatment of PA can be cost-effective, even when accounting for the upfront costs of cross-sectional imaging and AVS.^18^ While the focus of this investigation is the prescription cost savings and does not account for the cost of adrenalectomy, studies examining lifetime costs have shown that surgery is the most cost-effective strategy.^14,17^ For example, Sywak et al. found that among 24 patients who underwent adrenalectomy for PA, the mean estimated lifetime cost savings compared with medical therapy was CAD $31,132.^17^ Similarly, a decision analysis using a Markov State transition model found that for a patient with 41 remaining years of life, the cost of adrenalectomy would have to increase by 156%—from US $8,784 to US $22,525—for medical management to be more cost effective.^14^The decreased need for potassium supplementation among surgically treated patients, consistent with prior studies, represents an additional source of medication-related cost savings.^19^ Our results and those of past research are intuitive, given the wealth of data supporting the cost effectiveness of improved blood pressure control.^20,21^

Of note, our findings were consistent in the subgroup of individuals with resistant hypertension—a particularly high-risk and high-cost population. Current guidelines strongly recommend screening for PA among individuals with resistant hypertension given their markedly elevated likelihood of disease compared with those who require fewer medications.^6^ However, resistant hypertension is also a strong predictor of persistent antihypertensive medication requirements following adrenalectomy, which would incur added costs compared with those without resistant hypertension.^22,23^ The fact that these individuals benefited similarly from a cost perspective compared with individuals on fewer medications strongly reinforces the cost-related benefits of adrenalectomy. By extension, these findings suggest that early identification and definitive management of PA translate to lifetime cost savings.

Beyond the direct costs, surgical management of PA has well-established clinical benefits with potential intangible cost benefits compared to medical therapy. While both adrenalectomy and medical therapy can improve glucose metabolism, reduce left ventricular mass, and preserve renal function, surgical treatment yields earlier and more significant improvements in cardiac remodeling, particularly when performed earlier in the disease course.^24–37^ Importantly, multiple studies have demonstrated reduced incidence of cerebrovascular disease and lower all-cause mortality in patients undergoing adrenalectomy compared with those receiving medical treatment.^19,27,38^ In addition, several studies have reported significant improvements in quality of life following adrenalectomy, particularly among female patients, with one study noting higher scores for depression and anxiety in the medical management group.^39–42^ Beyond measurable clinical outcomes, surgical management confers additional meaningful benefits, as patients treated with adrenalectomy require fewer follow-up visits and clinic encounters, contributing to cost savings not captured in our analysis.^43^

This study is not without limitations, primarily related to the dataset. First and foremost, we are only able to follow patients with continuous insurance coverage periods. This dataset also does not capture uninsured patients, or those with Medicaid, which may contribute to bias in reported costs and inherent differences in patient populations. While we were able to identify more than 25,000 patients with an incident PA diagnosis, the subsequent rates of AVS were lower than expected—this may be due to coding inconsistencies, low rates of appropriate diagnostic work-up, or our strict requirements for continuous insurance coverage from incident diagnosis to completed work-up. We also noted significant attrition over the 1-year follow-up period. Similarly, the use of diagnostic codes remains a study limitation. While the requirement for AVS should ensure that most of the included patients truly had PA, without access to renin and aldosterone levels, we cannot fully confirm the biochemical diagnosis of PA in each individual subject.

Comparing medical versus surgical management poses a limitation in the timing of the “index” date. Surgical patients inherently have a delayed index date, in that they must complete AVS and then a separate procedure compared with medically managed patients. While one may anticipate that they would thus accumulate worse hypertensive disease, we did not demonstrate a difference in number of antihypertensive medications prescribed at index; this may be in part due to our strict requirement for completion of adrenalectomy within 1 year of AVS. Additionally, there may be inherent differences in the disease processes and outcomes between medically and surgically managed patients; medically managed patients may have comorbidities precluding surgery, or there may be inherent differences in hypertensive and cost outcomes between patients with bilateral adrenal hyperplasia and unilateral adenoma. Finally, we are unable to capture specific medication dosages in this dataset and thus are unable to account for improvements in dose requirements.

## Conclusions

Patients with PA who undergo adrenalectomy demonstrate significantly reduced antihypertensive medication compared with medically managed patients. While there is a significant upfront cost to surgical intervention, reduced long-term prescription costs are realized. Further research will be required to establish lifetime decreases in antihypertensive medication use and overall cost savings secondary to reduced prescription burden and comorbid conditions.

## Supplementary Information

Below is the link to the electronic supplementary material.

Supplementary file 1 (DOCX 66 KB)
