# Supplementary material for: Fewer Pills, Lower Bills: Antihypertensive and Cost Outcomes of Adrenalectomy in Primary Aldosteronism
Source: Ann Surg Oncol. 2026 Mar 4;33(6):5659–68. doi: 10.1245/s10434-026-19347-0 (PMC13179191; doi:10.1245/s10434-026-19347-0)
Supplement: Supplementary file 1 — (DOCX 66 KB) [file 10434_2026_19347_MOESM1_ESM.docx]

**SUPPLEMENT 1.** STROBE Reporting Guidelines Checklist

|  | | Item No | Recommendation | Page No |
| --- | --- | --- | --- | --- |
| **Title and abstract** | | 1 | (*a*) Indicate the study’s design with a commonly used term in the title or the abstract | 1-3 |
|  |  |  | (*b*) Provide in the abstract an informative and balanced summary of what was done and what was found |  |
| Introduction | | | | |
| Background/rationale | | 2 | Explain the scientific background and rationale for the investigation being reported | 4 |
| Objectives | | 3 | State specific objectives, including any prespecified hypotheses | 4 |
| Methods | | | | |
| Study design | | 4 | Present key elements of study design early in the paper | 4-6 |
| Setting | | 5 | Describe the setting, locations, and relevant dates, including periods of recruitment, exposure, follow-up, and data collection | 5 |
| Participants | | 6 | (*a*) Give the eligibility criteria, and the sources and methods of selection of participants. Describe methods of follow-up | 4-5 |
|  |  |  | (*b*) For matched studies, give matching criteria and number of exposed and unexposed |  |
| Variables | | 7 | Clearly define all outcomes, exposures, predictors, potential confounders, and effect modifiers. Give diagnostic criteria, if applicable | 5-6 |
| Data sources/ measurement | | 8* | For each variable of interest, give sources of data and details of methods of assessment (measurement). Describe comparability of assessment methods if there is more than one group | 4-6 |
| Bias | | 9 | Describe any efforts to address potential sources of bias | 6 |
| Study size | | 10 | Explain how the study size was arrived at | 5 |
| Quantitative variables | | 11 | Explain how quantitative variables were handled in the analyses. If applicable, describe which groupings were chosen and why | 5-6 |
| Statistical methods | | 12 | (*a*) Describe all statistical methods, including those used to control for confounding |  |
|  |  |  | (*b*) Describe any methods used to examine subgroups and interactions | 5-6 |
|  |  |  | (*c*) Explain how missing data were addressed |  |
|  |  |  | (*d*) If applicable, explain how loss to follow-up was addressed |  |
|  |  |  | (*e*) Describe any sensitivity analyses |  |
| Results | | | |  |
| Participants | | 13* | (a) Report numbers of individuals at each stage of study—eg numbers potentially eligible, examined for eligibility, confirmed eligible, included in the study, completing follow-up, and analysed | 6 |
|  |  |  | (b) Give reasons for non-participation at each stage |  |
|  |  |  | (c) Consider use of a flow diagram |  |
| Descriptive data | | 14* | (a) Give characteristics of study participants (eg demographic, clinical, social) and information on exposures and potential confounders | 6-8 |
|  |  |  | (b) Indicate number of participants with missing data for each variable of interest |  |
|  |  |  | (c) Summarise follow-up time (eg, average and total amount) |  |
| Outcome data | | 15* | Report numbers of outcome events or summary measures over time | 6-8 |
| Main results | 16 | (*a*) Give unadjusted estimates and, if applicable, confounder-adjusted estimates and their precision (eg, 95% confidence interval). Make clear which confounders were adjusted for and why they were included | | 6-8 |
|  |  | (*b*) Report category boundaries when continuous variables were categorized | |  |
|  |  | (*c*) If relevant, consider translating estimates of relative risk into absolute risk for a meaningful time period | |  |
| Other analyses | 17 | Report other analyses done—eg analyses of subgroups and interactions, and sensitivity analyses | | 7-8 |
| Discussion | | | | |
| Key results | 18 | Summarise key results with reference to study objectives | | 8 |
| Limitations | 19 | Discuss limitations of the study, taking into account sources of potential bias or imprecision. Discuss both direction and magnitude of any potential bias | | 9-10 |
| Interpretation | 20 | Give a cautious overall interpretation of results considering objectives, limitations, multiplicity of analyses, results from similar studies, and other relevant evidence | | 8-9 |
| Generalisability | 21 | Discuss the generalisability (external validity) of the study results | | 8-9 |
| Other information | | | | |
| Funding | 22 | Give the source of funding and the role of the funders for the present study and, if applicable, for the original study on which the present article is based | | 11 |

*Give information separately for exposed and unexposed groups.

**Note:** An Explanation and Elaboration article discusses each checklist item and gives methodological background and published examples of transparent reporting. The STROBE checklist is best used in conjunction with this article (freely available on the Web sites of PLoS Medicine at http://www.plosmedicine.org/, Annals of Internal Medicine at http://www.annals.org/, and Epidemiology at http://www.epidem.com/). Information on the STROBE Initiative is available at http://www.strobe-statement.org.

**SUPPLEMENT 2.** International Classification of Disease and Current Procedural Terminology codes utilized.

|  | **Included ICD codes** | **Included CPT codes** |
| --- | --- | --- |
| **Primary aldosteronism** | **ICD9:** 25510, 25511, 25512, 25514  **ICD10:** E260, E2601, E2602, E2609, E2689, E269 |  |
| **Adrenal vein sampling** |  | 36500, 75840, 75842, 75893 |
| **Adrenalectomy** | **ICD9:** 0700, 0701, 0702, 0721, 0722, 0729, 073  **ICD10:** 0GB20ZX, 0GB20ZZ, 0GB23ZX, 0GB23ZZ, 0GB24ZX, 0GB24ZZ, 0GB30ZX, 0GB30ZZ, 0GB33ZX, 0GB33ZZ, 0GB34ZX, 0GB34ZZ, 0GB40ZX, 0GB40ZZ, 0GB43ZX, 0GB43ZZ, 0GB44ZX, 0GB44ZZ, 0GT20ZZ, 0GT24ZZ, 0GT30ZZ, 0GT34ZZ, 0GT40ZZ, 0GT44ZZ | 60540, 6545, 60650 |

**SUPPLEMENT 3.** Antihypertensive medications as divided by class. Combination drugs were counted for both classes of medication.

| **Drug Class** | **Medications Included** |
| --- | --- |
| Angiotensin converting enzyme inhibitors | AMLODIPINE BESYLATE/BENAZEPRIL, BENAZEPRIL HCL, BENAZEPRIL/HYDROCHLOROTHIAZIDE, CAPTOPRIL, CAPTOPRIL/HYDROCHLOROTHIAZIDE, ENALAPRIL MALEATE, ENALAPRIL MALEATE/FELODIPINE, ENALAPRIL MALEATE/HCTZ, ENALAPRIL/HYDROCHLOROTHIAZIDE, FOSINOPRIL SODIUM, FOSINOPRIL/HYDROCHLOROTHIAZIDE, LISINOPRIL, LISINOPRIL/DIETARY SUP.CMB10, LISINOPRIL/HYDROCHLOROTHIAZIDE, MOEXIPRIL HCL, MOEXIPRIL/HYDROCHLOROTHIAZIDE, PERINDOPRIL ARG/AMLODIPINE BES, PERINDOPRIL ERBUMINE, QUINAPRIL HCL, QUINAPRIL HCL/MAG CARB, QUINAPRIL/HCTZ/MAG CARB, QUINAPRIL/HYDROCHLOROTHIAZIDE, RAMIPRIL, TRANDOLAPRIL, TRANDOLAPRIL/VERAPAMIL HCL |
| Alpha-1 blockers | DOXAZOSIN MESYLATE, PRAZOSIN HCL, PRAZOSIN HCL/POLYTHIAZIDE, TERAZOSIN HCL |
| Angiotensin receptor blockers | ALISKIREN/VALSARTAN, AMLODIPINE BES/OLMESARTAN MED, AMLODIPINE BESYLATE/VALSARTAN, AMLODIPINE/VALSARTAN, AMLODIPINE/VALSARTAN/HCTHIAZID, AMLODIPINE/VALSARTAN/HCTZ, AZILSARTAN MED/CHLORTHALIDONE, AZILSARTAN MEDOXOMIL, CANDESARTAN CILEXETIL, CANDESARTAN CILEXETIL/HCTZ, CANDESARTAN/HYDROCHLOROTHIAZID, EPROSARTAN MESYLATE, EPROSARTAN/HYDROCHLOROTHIAZIDE, IRBESARTAN, IRBESARTAN/HYDROCHLOROTHIAZIDE, LOSARTAN POTASSIUM, LOSARTAN POTASSIUM/HCTZ, LOSARTAN/HYDROCHLOROTHIAZIDE, NEBIVOLOL HCL/VALSARTAN, OLMESARTAN MED/AMLODIPINE/HCTZ, OLMESARTAN MEDOXOMIL, OLMESARTAN/AMLODIPIN/HCTHIAZID, OLMESARTAN/HYDROCHLOROTHIAZIDE, OLMESARTN/HYDROCHLOROTHIAZIDE, SACUBITRIL/VALSARTAN, TELMISARTAN, TELMISARTAN/AMLODIPINE, TELMISARTAN/HYDROCHLOROTHIAZID, VALSARTAN, VALSARTAN/HYDROCHLOROTHIAZIDE |
| Beta blockers - cardioselective | ATENOLOL, ATENOLOL/CHLORTHALIDONE, BETAXOLOL HCL, BISOPROL/HYDROCHLOROTHIAZIDE, BISOPROLOL FUMARATE, BISOPROLOL FUMARATE/HCTZ, BISOPROLOL/HYDROCHLOROTHIAZIDE, METOPROL/HYDROCHLOROTHIAZIDE, METOPROLOL SU/HYDROCHLOROTHIAZ, METOPROLOL SUCCINATE, METOPROLOL SUCCINATE/HCTZ, METOPROLOL TARTRATE, METOPROLOL/DIETARY SUPPL.CMB10, METOPROLOL/HYDROCHLOROTHIAZIDE |
| Beta blockers – cardioselective and vasodilatory | NEBIVOLOL HCL, NEBIVOLOL HCL/VALSARTAN |
| Beta blockers – combined alpha and beta-receptor | CARVEDILOL, CARVEDILOL PHOSPHATE, LABETALOL HCL |
| Beta blockers – intrinsic sympathomimetic activity | ACEBUTOLOL HCL, PENBUTOLOL SULFATE, PINDOLOL |
| Beta blockers - noncardioselective | NADOLOL, NADOLOL/BENDROFLUMETHIAZIDE, PROPRANOLOL HCL, PROPRANOLOL/HYDROCHLOROTHIAZID |
| Calcium channel blockers – dihydropyridines | ALISKIREN/AMLODIPIN/HCTHIAZIDE, ALISKIREN/AMLODIPINE, ALISKIREN/AMLODIPINE BESYLATE, ALISKIREN/AMLODIPINE/HCTZ, AMLODIPINE BENZOATE, AMLODIPINE BES/OLMESARTAN MED, AMLODIPINE BESYLATE, AMLODIPINE BESYLATE/BENAZEPRIL, AMLODIPINE BESYLATE/CELECOXIB, AMLODIPINE BESYLATE/VALSARTAN, AMLODIPINE/ATORVAST CAL, AMLODIPINE/ATORVASTATIN, AMLODIPINE/VALSARTAN, AMLODIPINE/VALSARTAN/HCTHIAZID, AMLODIPINE/VALSARTAN/HCTZ, ENALAPRIL MALEATE/FELODIPINE, FELODIPINE, ISRADIPINE, NICARDIPINE HCL, NIFEDIPINE, NISOLDIPINE, OLMESARTAN MED/AMLODIPINE/HCTZ, OLMESARTAN/AMLODIPIN/HCTHIAZID, PERINDOPRIL ARG/AMLODIPINE BES, TELMISARTAN/AMLODIPINE |
| Calcium channel blockers – nondihydropyridines | DILTIAZEM HCL, TRANDOLAPRIL/VERAPAMIL HCL, VERAPAMIL HCL |
| Central alpha-2 agonists and other centrally acting | CLONIDINE, CLONIDINE HCL, CLONIDINE HCL/CHLORTHALIDONE, GUANFACINE HCL, METHYLDOPA, METHYLDOPA/HYDROCHLOROTHIAZIDE |
| Direct renin inhibitors | ALISKIREN HEMIFUMARATE, ALISKIREN/AMLODIPIN/HCTHIAZIDE, ALISKIREN/AMLODIPINE, ALISKIREN/AMLODIPINE BESYLATE, ALISKIREN/AMLODIPINE/HCTZ, ALISKIREN/HYDROCHLOROTHIAZIDE, ALISKIREN/VALSARTAN |
| Direct vasodilators | HYDRALAZINE HCL, HYDRALAZINE/HYDROCHLOROTHIAZID, HYDRALAZINE/RESERPIN/HCTZ, ISOSORB DINIT/HYDRALAZINE HCL, ISOSORBIDE DINIT/HYDRALAZINE, MINOXIDIL |
| Diuretics - loop | BUMETANIDE, FUROSEMIDE, TORSEMIDE |
| Diuretics – aldosterone antagonists | EPLERENONE, SPIRONOLACT/HYDROCHLOROTHIAZID, SPIRONOLACTONE, SPIRONOLACTONE, MICRONIZED, SPIRONOLACTONE/HCTZ |
| Diuretics – potassium sparing | AMILORIDE HCL, AMILORIDE HCL/HCTZ, AMILORIDE/HYDROCHLOROTHIAZIDE, TRIAMTERENE, TRIAMTERENE/HCTZ, TRIAMTERENE/HYDROCHLOROTHIAZIDE |
| Thiazide or thiazide-type diuretics | ALISKIREN/AMLODIPIN/HCTHIAZIDE, ALISKIREN/AMLODIPINE/HCTZ, ALISKIREN/HYDROCHLOROTHIAZIDE, AMILORIDE HCL/HCTZ, AMILORIDE/HYDROCHLOROTHIAZIDE, AMLODIPINE/VALSARTAN/HCTHIAZID, AMLODIPINE/VALSARTAN/HCTZ, ATENOLOL/CHLORTHALIDONE, AZILSARTAN MED/CHLORTHALIDONE, BENAZEPRIL/HYDROCHLOROTHIAZIDE, BISOPROL/HYDROCHLOROTHIAZIDE, BISOPROLOL FUMARATE/HCTZ, BISOPROLOL/HYDROCHLOROTHIAZIDE, CANDESARTAN CILEXETIL/HCTZ, CANDESARTAN/HYDROCHLOROTHIAZID, CAPTOPRIL/HYDROCHLOROTHIAZIDE, CHLORTHALIDONE, CLONIDINE HCL/CHLORTHALIDONE, ENALAPRIL MALEATE/HCTZ, ENALAPRIL/HYDROCHLOROTHIAZIDE, EPROSARTAN/HYDROCHLOROTHIAZIDE, FOSINOPRIL/HYDROCHLOROTHIAZIDE, HYDRALAZINE/HYDROCHLOROTHIAZID, HYDRALAZINE/RESERPIN/HCTZ, HYDROCHLOROTHIAZIDE, INDAPAMIDE, IRBESARTAN/HYDROCHLOROTHIAZIDE, LISINOPRIL/HYDROCHLOROTHIAZIDE, LOSARTAN POTASSIUM/HCTZ, LOSARTAN/HYDROCHLOROTHIAZIDE, METHYLDOPA/HYDROCHLOROTHIAZIDE, METOLAZONE, METOPROL/HYDROCHLOROTHIAZIDE, METOPROLOL SU/HYDROCHLOROTHIAZ, METOPROLOL SUCCINATE/HCTZ, METOPROLOL/HYDROCHLOROTHIAZIDE, MOEXIPRIL/HYDROCHLOROTHIAZIDE, OLMESARTAN MED/AMLODIPINE/HCTZ, OLMESARTAN/AMLODIPIN/HCTHIAZID, OLMESARTAN/HYDROCHLOROTHIAZIDE, OLMESARTN/HYDROCHLOROTHIAZIDE, PROPRANOLOL/HYDROCHLOROTHIAZID, QUINAPRIL/HCTZ/MAG CARB, QUINAPRIL/HYDROCHLOROTHIAZIDE, SPIRONOLACT/HYDROCHLOROTHIAZID, SPIRONOLACTONE/HCTZ, TELMISARTAN/HYDROCHLOROTHIAZID, TIMOLOL/HYDROCHLOROTHIAZIDE, TRIAMTERENE/HCTZ, TRIAMTERENE/HYDROCHLOROTHIAZID, VALSARTAN/HYDROCHLOROTHIAZIDE |

**SUPPLEMENT 4.** Potassium supplements included in sub-analysis.

| **Potassium Supplements Generic Names** |
| --- |
| POTASSIUM CHLORIDE, POTASSIUM GLUCONATE, POTASSIUM CITRATE, POTASSIUM PHOSPHATE, POTASSIUM BICARBONATE |

**SUPPLEMENT 5**. Trends in median cumulative prescription costs from pre- to post-index date.

| **Time Period** | **Overall Cohort** | | **Medically managed** | | **Adrenalectomy** | | **P-value^a^** |
| --- | --- | --- | --- | --- | --- | --- | --- |
|  | **n** | **Median (Q1–Q3)** | **n** | **Median (Q1–Q3)** | **n** | **Median (Q1–Q3)** |  |
| **Prior to index date** |  |  |  |  |  |  |  |
| -6 months | 911 | 313.79 (89.08-1,006.31) | 439 | 293.15 (70.82-1,031.91) | 472 | 358.93 (100.92-991.62) | 0.242 |
| -3 months | 911 | 145.64 (37.50-484.81) | 439 | 143.59 (33.00-459.15) | 472 | 152.87 (41.29-507.74) | 0.351 |
| -1 month | 911 | 28.29 (0.00-129.94) | 439 | 28.73 (0.00-139.38) | 472 | 27.31 (0.00-120.10) | 0.744 |
| **After index date** |  |  |  |  |  |  |  |
| +3 months | 911 | 92.04 (13.14-333.75) | 439 | 169.61 (46.88-546.71) | 472 | 36.36 (0.00-184.91) | **<0.001** |
| +6 months | 826 | 168.94 (25.54-709.30) | 393 | 367.59 (106.16-1,119.14) | 433 | 71.65 (6.64-337.08) | **<0.001** |
| +9 months | 735 | 292.43 (40.94-1,041.93) | 342 | 599.93 (165.95-1,614.32) | 393 | 109.15 (10.52-481.39) | **<0.001** |
| +12 months | 653 | 396.48 (56.82-1,388.50) | 306 | 829.63 (245.94-2,084.53) | 347 | 161.13 (14.73-688.91) | **<0.001** |

a = p-value compares medically managed versus adrenalectomy patients

**SUPPLEMENT 6.** Trends in binary utilization of potassium supplementation from pre- to post-index date.

| **Time Period** | **Overall Cohort** | | **Medically managed** | | **Adrenalectomy** | | **P-value^a^** |
| --- | --- | --- | --- | --- | --- | --- | --- |
|  | **N** | **Prescribed K,**  **n (%)** | **N** | **Prescribed K,**  **n (%)** | **N** | **Prescribed K,**  **n (%)** |  |
| **Prior to index date** |  |  |  |  |  |  |  |
| -12 to -9 months | 782 | 337 (43.1%) | 377 | 132 (35.0%) | 405 | 205 (50.6%) | **<0.001** |
| -9 to -6 months | 844 | 394 (46.7%) | 413 | 153 (37.0%) | 431 | 241 (55.9%) | **<0.001** |
| -6 to -3 months | 911 | 476 (52.3%) | 439 | 183 (41.7%) | 472 | 293 (62.1%) | **<0.001** |
| -3 to 0 months | 911 | 476 (52.3%) | 439 | 195 (44.4%) | 472 | 281 (59.5%) | **<0.001** |
| **After index date** |  |  |  |  |  |  |  |
| 0 to +3 months | 911 | 223 (24.5%) | 439 | 159 (36.2%) | 472 | 64 (13.6%) | **<0.001** |
| +3 to +6 months | 826 | 118 (14.3%) | 393 | 96 (24.4%) | 433 | 22 (5.1%) | **<0.001** |
| +6 to +9 months | 735 | 89 (12.1%) | 342 | 68 (19.9%) | 393 | 21 (5.3%) | **<0.001** |
| +9 to +12 months | 653 | 82 (12.6%) | 306 | 66 (21.6%) | 347 | 16 (4.6%) | **<0.001** |

a = p-value compares medically managed versus adrenalectomy patients

**SUPPLEMENT 7**. Multivariable logistic regression modeling analyzing predictors of potassium supplementation at 12 months.

| **Covariate** | **Odds Ratio** | **95% CI** | **P-value** |
| --- | --- | --- | --- |
| **Age, per year** | 1.02 | (0.99, 1.05) | 0.115 |
| **Male sex** | 1.42 | (0.81, 2.48) | 0.219 |
| **Race/Ethnicity (ref: White)** |  |  |  |
| Asian | 0.33 | (0.04, 2.67) | 0.297 |
| Black | 1.41 | (0.72, 2.76) | 0.321 |
| Hispanic | 0.45 | (0.16, 1.23) | 0.120 |
| Unknown | 0.80 | (0.21, 3.09) | 0.745 |
| **Household income (ref: $100K+)** |  |  |  |
| <$40K | 1.63 | (0.74, 3.62) | 0.227 |
| $40K-$49K | 2.33 | (0.86, 6.31) | 0.097 |
| $50K-$59K | 1.27 | (0.42, 3.77) | 0.672 |
| $60K-$74K | 0.86 | (0.30, 2.44) | 0.774 |
| $75K-$99K | 1.15 | (0.50, 2.64) | 0.749 |
| Unknown | 1.25 | (0.49, 3.18) | 0.636 |
| **Medicare insurance (ref: Commercial)** | 0.61 | (0.28, 1.31) | 0.205 |
| **Elixhauser score, per point** | 1.06 | (0.96, 1.17) | 0.233 |
| **Prescribed K supplementation at baseline** | 4.05 | (2.27, 7.21) | **<0.001** |
| **Baseline number of AHM, per AHM** | 0.87 | (0.72, 1.04) | 0.127 |
| **Baseline monthly AHM cost, per $** | 1.00 | (1.00, 1.00) | 0.357 |
| **Adrenalectomy** | 0.13 | (0.07, 0.24) | **<0.001** |

**SUPPLEMENT 8.** Baseline demographic characteristics amongst subcohort of patients with resistant hypertension compared by medical versus surgical management.

| **Variable** | **Overall cohort (n=534)** | **Medically managed (n=251)** | **Adrenalectomy (n=283)** | **P-value^b^** |
| --- | --- | --- | --- | --- |
| **Median age, years (IQR)** | 57.0 (49.0-65.0) | 58.0 (51.0-65.0) | 57.0 (49.0-65.0) | **0.009** |
| **Sex** |  |  |  |  |
| Female | 170 (31.8%) | 84 (33.5%) | 86 (30.4%) | 0.446 |
| Male | 364 (68.2%) | 167 (66.5%) | 197 (69.6%) |  |
| **Race** |  |  |  |  |
| Asian | 18 (3.4%) | 7 (2.8%) | 11 (3.9%) | 0.348 |
| Black | 105 (19.7%) | 53 (21.1%) | 52 (18.4%) |  |
| Hispanic | 41 (7.7%) | 24 (9.6%) | 17 (6.0%) |  |
| White | 343 (64.2%) | 157 (62.5%) | 186 (65.7%) |  |
| Unknown | 27 (5.1%) | 10 (4.0%) | 17 (6.0%) |  |
| **Education level** |  |  |  |  |
| Less than 12th Grade | N<5 | N<5 | N<5 | 0.782 |
| High School Diploma | 119 (22.3%) | 54 (21.5%) | 65 (23.0%) |  |
| Less than Bachelor Degree | 291 (54.5%) | 142 (56.6%) | 149 (52.7%) |  |
| Bachelor Degree Plus | 106 (19.9%) | 48 (19.1%) | 58 (20.5%) |  |
| Unknown | 17 (3.2%) | 7 (2.8%) | 10 (3.5%) |  |
| **Household income** |  |  |  |  |
| <$40K | 100 (18.7%) | 42 (16.7%) | 58 (20.5%) | 0.744 |
| $40K-$49K | 22 (4.1%) | 11 (4.4%) | 11 (3.9%) |  |
| $50K-$59K | 32 (6.0%) | 17 (6.8%) | 15 (5.3%) |  |
| $60K-$74K | 54 (10.1%) | 25 (10.0%) | 29 (10.2%) |  |
| $75K-$99K | 83 (15.5%) | 45 (17.9%) | 38 (13.4%) |  |
| $100K+ | 174 (32.6%) | 79 (31.5%) | 95 (33.6%) |  |
| Unknown | 69 (12.9%) | 32 (12.7%) | 37 (13.1%) |  |
| **Insurance status** |  |  |  |  |
| Commercial | 394 (73.8%) | 181 (72.1%) | 213 (75.3%) | 0.408 |
| Medicare | 140 (26.2%) | 70 (27.9%) | 70 (24.7%) |  |
| **Elixhauser score** |  |  |  |  |
| Median (IQR) | 2.0 (0.0-4.0) | 0.0 (0.0-3.0) | 2.0 (0.0-4.0) | **<0.001** |
| Mean (SD) | 2.4 (2.8) | 1.8 (2.8) | 3.0 (2.7) | **<0.001** |

a = age at index date

b = p-value compares medically managed versus adrenalectomy patients

**SUPPLEMENT 9.** Trends in mean number of prescribed antihypertensive medication classes from pre- to post-index date in subcohort of patients with resistant hypertension.

| **Time Period** | **Overall Cohort** | | **Medically managed** | | **Adrenalectomy** | | **P-value^a^** |
| --- | --- | --- | --- | --- | --- | --- | --- |
|  | **n** | **Mean (SD)** | **n** | **Mean (SD)** | **n** | **Mean (SD)** |  |
| **Prior to index date** |  |  |  |  |  |  |  |
| -12 to -9 months | 455 | 3.3 (1.5) | 218 | 3.0 (1.5) | 237 | 3.5 (1.5) | **0.003** |
| -9 to -6 months | 492 | 3.4 (1.5) | 239 | 3.3 (1.5) | 253 | 3.6 (1.4) | **0.034** |
| -6 to -3 months | 534 | 3.6 (1.4) | 251 | 3.5 (1.5) | 283 | 3.7 (1.4) | 0.156 |
| -3 to 0 months | 534 | 4.0 (1.0) | 251 | 4.0 (1.0) | 283 | 3.9 (1.0) | 0.680 |
| **After index date** |  |  |  |  |  |  |  |
| 0 to +3 months | 534 | 3.0 (1.5) | 251 | 3.7 (1.3) | 283 | 2.3 (1.5) | **<0.001** |
| +3 to +6 months | 485 | 2.7 (1.5) | 224 | 3.4 (1.3) | 261 | 2.0 (1.5) | **<0.001** |
| +6 to +9 months | 435 | 2.6 (1.6) | 197 | 3.3 (1.4) | 238 | 2.0 (1.4) | **<0.001** |
| +9 to +12 months | 390 | 2.5 (1.5) | 178 | 3.3 (1.2) | 212 | 1.9 (1.5) | **<0.001** |

a = p-value compares medically managed versus adrenalectomy patients

**SUPPLEMENT 10**. Multivariable linear regression modeling analyzing predictors of increased antihypertensive burden after 12 months in subcohort of patients with resistant hypertension.

| **Covariate** | **Coefficient** | **95% CI** | **P-value** |
| --- | --- | --- | --- |
| **Age, per year** | 0.03 | (0.01, 0.04) | **<0.001** |
| **Male sex** | 0.53 | (0.24, 0.82) | **<0.001** |
| **Race/Ethnicity (ref: White)** |  |  |  |
| Asian | 0.15 | (-0.62, 0.93) | 0.701 |
| Black | 0.40 | (0.05, 0.75) | **0.025** |
| Hispanic | 0.11 | (-0.37, 0.58) | 0.654 |
| Unknown | -0.06 | (-0.69, 0.57) | 0.847 |
| **Household Income (ref: $100K+)** |  |  |  |
| <$40K | 0.04 | (-0.37, 0.45) | 0.852 |
| $40K-$49K | -0.32 | (-0.99, 0.35) | 0.347 |
| $50K-$59K | 0.27 | (-0.31, 0.85) | 0.360 |
| $60K-$74K | 0.15 | (-0.29, 0.60) | 0.504 |
| $75K-$99K | -0.19 | (-0.60, 0.22) | 0.352 |
| Unknown | 0.26 | (-0.23, 0.74) | 0.298 |
| **Medicare insurance (ref: Commercial)** | -0.32 | (-0.70, 0.07) | 0.103 |
| **Elixhauser score, per point** | 0.01 | (-0.04, 0.06) | 0.612 |
| **Baseline number of AHM, per AHM** | 0.34 | (0.21, 0.47) | **<0.001** |
| **Adrenalectomy** | -1.35 | (-1.62, -1.08) | **<0.001** |

**SUPPLEMENT 11**. Trends in median cumulative prescription costs from pre- to post-index date in the subcohort of patients with resistant hypertension.

| **Time Period** | **Overall Cohort** | | **Medically managed** | | **Adrenalectomy** | | **P-value^a^** |
| --- | --- | --- | --- | --- | --- | --- | --- |
|  | **n** | **Median (Q1–Q3)** | **n** | **Median (Q1–Q3)** | **n** | **Median (Q1–Q3)** |  |
| **Prior to index date** |  |  |  |  |  |  |  |
| -6 months | 534 | 622.52 (222.11-1,353.56) | 251 | 656.05 (219.32-1,340.42) | 283 | 609.33 (222.11-1,362.35) | 0.809 |
| -3 months | 534 | 306.49 (106.63-732.79) | 251 | 315.17 (113.45-713.92) | 283 | 288.29 (102.12-759.35) | 0.994 |
| -1 month | 534 | 63.02 (12.52-196.82) | 251 | 72.46 (14.46-220.50) | 283 | 56.52 (11.84-187.68) | 0.187 |
| **After index date** |  |  |  |  |  |  |  |
| +3 months | 534 | 154.85 (38.12-481.39) | 251 | 299.47 (105.52-709.30) | 283 | 67.16 (17.29-251.64) | **<0.001** |
| +6 months | 485 | 291.47 (80.95-984.36) | 224 | 708.08 (247.21-1,372.16) | 261 | 122.92 (33.29-469.58) | **<0.001** |
| +9 months | 435 | 451.54 (125.06-1,439.54) | 197 | 912.10 (367.96-1,940.16) | 238 | 200.06 (50.20-705.55) | **<0.001** |
| +12 months | 390 | 616.95 (170.04-1,870.95) | 178 | 1,335.95 (526.77-2,520.12) | 212 | 326.03 (67.61-918.89) | **<0.001** |

a = p-value compares medically managed versus adrenalectomy patients

**SUPPLEMENT 12**. Multivariable linear regression modeling analyzing predictors of increased prescription costs over 12 months in subcohort of patients with resistant hypertension.

| **Covariate** | **Coefficient** | **95% CI** | **P-value** |
| --- | --- | --- | --- |
| **Age, per year** | 8.79 | (-7.89, 25.47) | 0.301 |
| **Male sex** | 660.73 | (323.65, 997.80) | **<0.001** |
| **Race/Ethnicity (ref: White)** |  |  |  |
| Asian | -435.85 | (-1345.00, 473.30) | 0.347 |
| Black | 330.29 | (-77.37, 737.94) | 0.112 |
| Hispanic | -253.75 | (-809.83, 302.33) | 0.370 |
| Unknown | -363.23 | (-1095.37, 368.91) | 0.330 |
| **Household income (ref: $100K+)** |  |  |  |
| <$40K | -104.96 | (-581.58, 371.66) | 0.665 |
| $40K-$49K | -373.07 | (-1162.01, 415.87) | 0.353 |
| $50K-$59K | -103.10 | (-779.74, 573.53) | 0.765 |
| $60K-$74K | -145.27 | (-667.63, 377.08) | 0.585 |
| $75K-$99K | 3.87 | (-475.73, 483.47) | 0.987 |
| Unknown | 302.84 | (-262.10, 867.78) | 0.293 |
| **Medicare insurance (ref: Commercial)** | -295.60 | (-746.81, 155.61) | 0.199 |
| **Elixhauser score, per point** | 11.40 | (-47.10, 69.89) | 0.702 |
| **Baseline monthly AHM cost, per $** | 2.84 | (2.23, 3.44) | **<0.001** |
| **Adrenalectomy** | -1050.60 | (-1366.63, -734.56) | **<0.001** |
